# Supplementary material for: MetaQTL: a package of new computational methods for the meta-analysis of QTL mapping experiments
Source: BMC Bioinformatics. 2007 Feb 8;8:49. doi: 10.1186/1471-2105-8-49 (PMC1808479; doi:10.1186/1471-2105-8-49)
Supplement: Additional File 7 — MetaQTL Package : jar file and tutorial. This Zip archive contains both the MetaQTL JAR file and the files of the tutorial. [file 1471-2105-8-49-S7.zip › org.inra.metaqtl/doc/org/metaqtl/bio/entity/factory/MapTabFactory.html]

MapTabFactory


|  |  |  |
| --- | --- | --- |
| **Overview** \| **Package** \| **Class** \| **Use** \| **Tree** \| **Deprecated** \| **Index** \| **Help** | |  |
| **PREV CLASS**   **NEXT CLASS** | **FRAMES**    **NO FRAMES**     **All Classes** |  |
| SUMMARY: NESTED \| FIELD \| CONSTR \| METHOD | DETAIL: FIELD \| CONSTR \| METHOD |  |


---


## org.metaqtl.bio.entity.factory Class MapTabFactory

```
java.lang.Object
  org.metaqtl.bio.entity.factory.BioEntityFactory
      org.metaqtl.bio.entity.factory.MapTabFactory
```

---

``` public class MapTabFactory extends BioEntityFactory ```

**Author:**
:   Jean-Baptiste

---

**Constructor Summary**

`MapTabFactory()`


| **Method Summary** | |
| --- | --- |
| `IBioEntity` | `load(java.io.InputStream stream)` |
| `IBioEntity` | `load(java.io.Reader reader)` |
| `void` | `unload(IBioEntity entity, java.io.OutputStream stream)` |
| `void` | `unload(IBioEntity obj, java.io.Writer writer)` |

**Methods inherited from class java.lang.Object**

`clone, equals, finalize, getClass, hashCode, notify, notifyAll, toString, wait, wait, wait`

**Constructor Detail**

### MapTabFactory

```
public MapTabFactory()
```


**Method Detail**

### unload

```
public void unload(IBioEntity obj,
                   java.io.Writer writer)
            throws java.io.IOException
```

:   **Specified by:**: `unload` in class `BioEntityFactory`
:   **Throws:**: `java.io.IOException`

---


### load

```
public IBioEntity load(java.io.Reader reader)
                throws java.io.IOException
```

:   **Specified by:**: `load` in class `BioEntityFactory`
:   **Throws:**: `java.io.IOException`

---


### load

```
public IBioEntity load(java.io.InputStream stream)
                throws java.io.IOException
```

:   **Specified by:**: `load` in class `BioEntityFactory`
:   **Throws:**: `java.io.IOException`

---


### unload

```
public void unload(IBioEntity entity,
                   java.io.OutputStream stream)
            throws java.io.IOException
```

:   **Specified by:**: `unload` in class `BioEntityFactory`
:   **Throws:**: `java.io.IOException`


---


|  |  |  |
| --- | --- | --- |
| **Overview** \| **Package** \| **Class** \| **Use** \| **Tree** \| **Deprecated** \| **Index** \| **Help** | |  |
| **PREV CLASS**   **NEXT CLASS** | **FRAMES**    **NO FRAMES**     **All Classes** |  |
| SUMMARY: NESTED \| FIELD \| CONSTR \| METHOD | DETAIL: FIELD \| CONSTR \| METHOD |  |


---
